# A Review of the Protective Effects of Ferula asafoetida on the Liver, Kidney, and Testes Against Formaldehyde-Induced Damage

**DOI:** 10.7759/cureus.79545

**Published:** 2025-02-24

**Authors:** Sravani Priya Thulluri, Karthick Selvaraj, Divya Prafulla Yerraguntla, S. Saravana Kumar

**Affiliations:** 1 Department of Anatomy, Meenakshi Medical College, Chennai, IND; 2 Department of Pathology, Maheshwara Medical College, Sangareddy, IND

**Keywords:** antioxidant and anti-inflammatory properties, ferula asafoetida, formaldehyde-induced toxicity, oxidative stress mitigation, pathophysiological mechanisms

## Abstract

Formaldehyde, a pervasive environmental toxin, has well-documented deleterious effects on critical organ systems. This review examines the therapeutic potential of *Ferula asafoetida* (FA) root extracts in protecting the testes, kidneys, and liver against formaldehyde-induced toxicity in rodent models. The literature reveals that FA's active constituents, known for their potent antioxidant and anti-inflammatory activities, may counteract oxidative stress and cellular damage caused by formaldehyde exposure. The review explores formaldehyde-induced pathophysiological mechanisms and FA's protective effects, including mitigation of oxidative damage, inflammation, and apoptosis. By analyzing empirical evidence, it compares the efficacy of various extract preparations, dosage regimens, and treatment durations. The review also addresses methodological heterogeneity and challenges in extrapolating findings to humans. It concludes with a call for rigorous, controlled clinical trials to validate FA's therapeutic viability, offering hope for those affected by formaldehyde toxicity.

## Introduction and background

**Figure 1:**
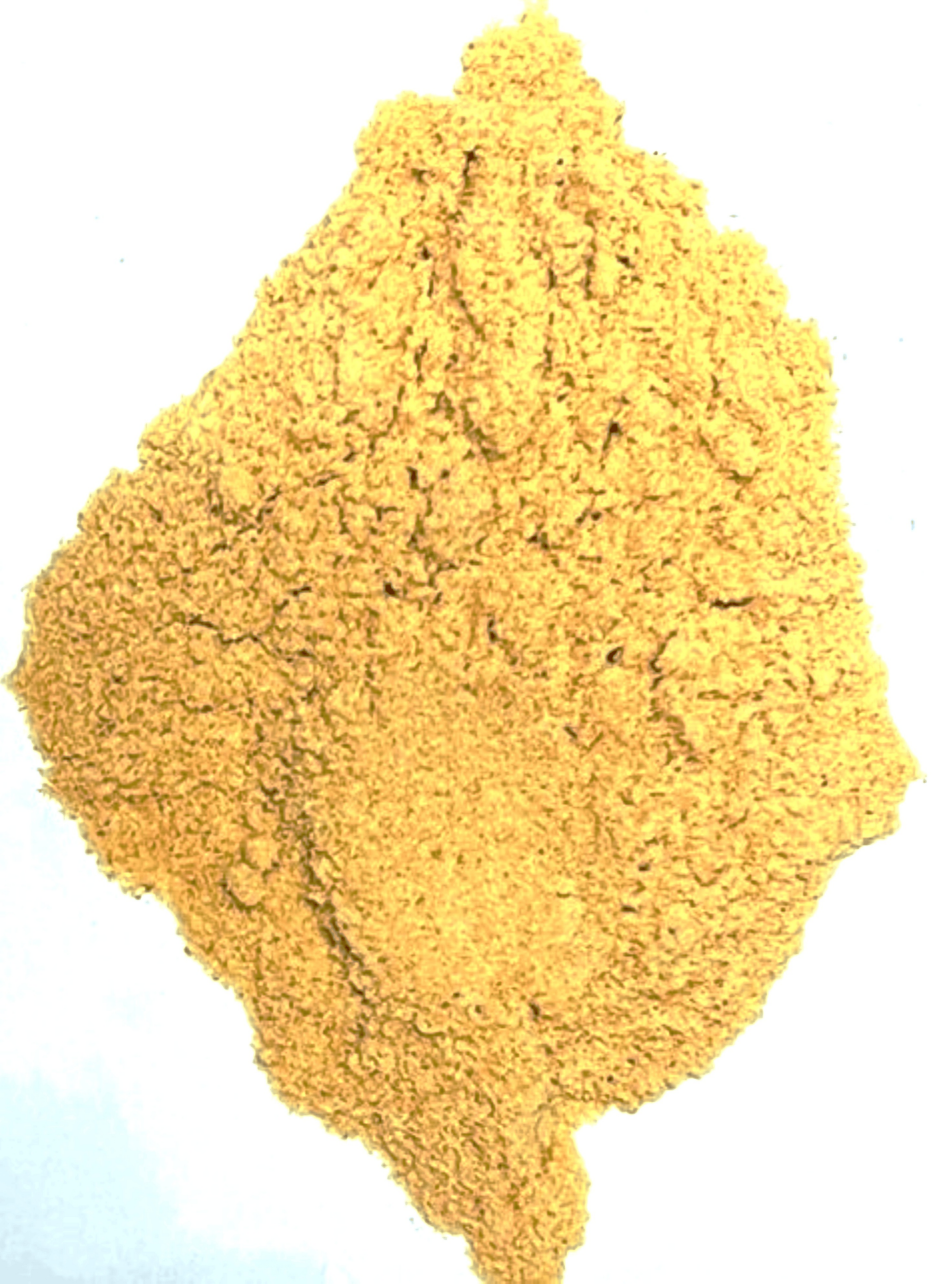
Asafoetida powder. The image was taken by the author.

Traditional medicine includes many amazing plants, and one of them is *Ferula asafoetida* (FA), also known as asafetida (Figure 1). This plant has a long history and is known for its healing properties. It comes from the roots of *Ferula* plants that grow in the Middle East. People have used it for a long time in different types of traditional medicine, such as Ayurveda, Unani, and Iranian folk medicine [1,2].

**Figure 2:**
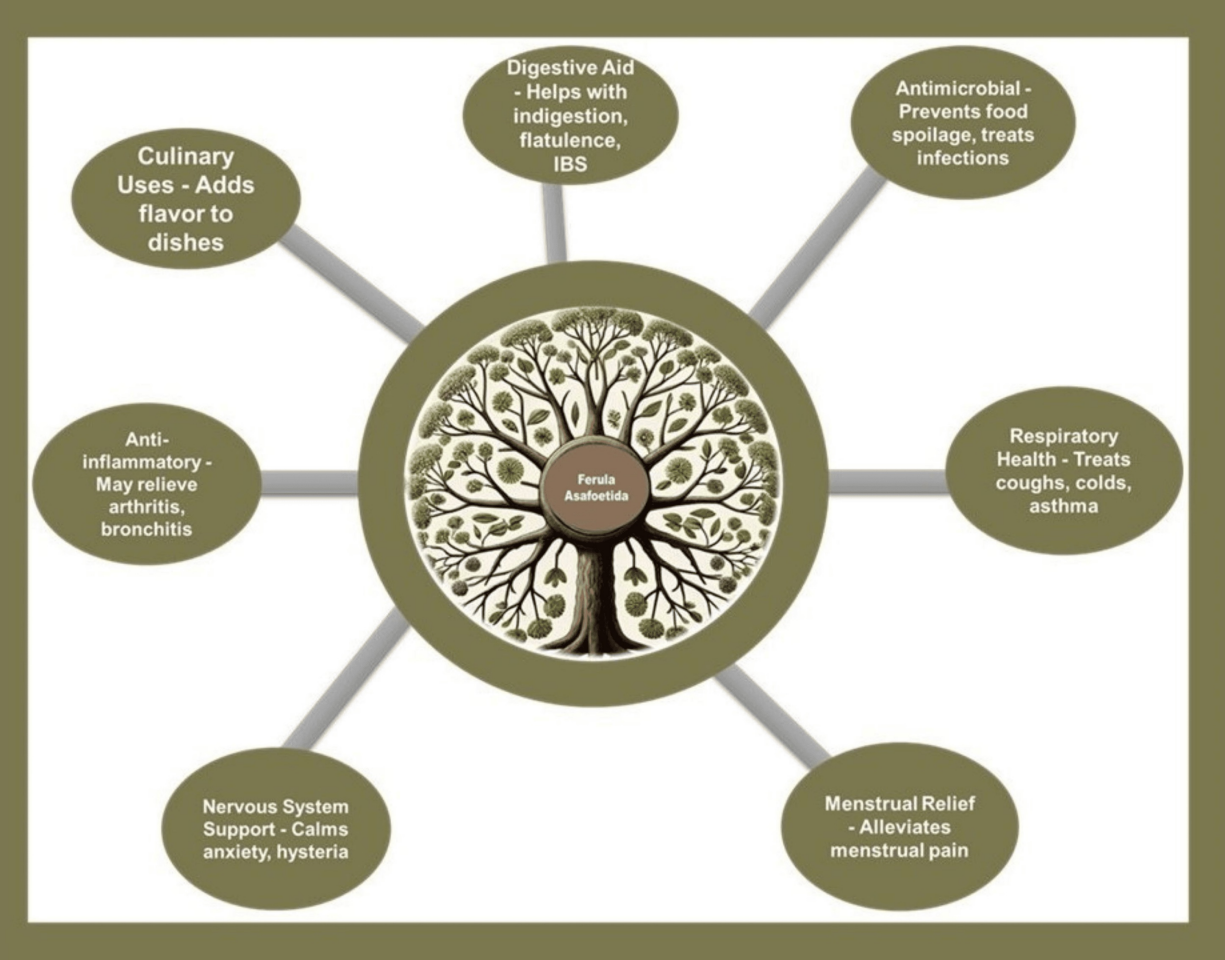
Benefits of Ferula asafoetida: Digestive aid, noting its help with indigestion, flatulence, and irritable bowel syndrome; antimicrobial properties, which prevent food spoilage and treat infections; anti-inflammatory benefits, potentially relieving arthritis and bronchitis; respiratory health, used for treating coughs, colds, and asthma; nervous system support, calming anxiety and hysteria; and menstrual relief, alleviating menstrual pain. The image was created using the ChatGPT AI tool (OpenAI, San Francisco, CA).

As a culinary spice and folk remedy, it is prized for its ability to impart a savory, umami-like flavor to dishes, especially vegetarian cuisine, where it acts as a flavor enhancer. Beyond its gustatory contributions, asafoetida is acclaimed for its health benefits; it serves as a digestive aid, easing symptoms like indigestion, bloating, and irritable bowel syndrome (IBS). Its antimicrobial properties make it valuable in preventing food spoilage and in treating microbial infections. Asafoetida is renowned for its anti-inflammatory properties, which help alleviate conditions like arthritis and bronchitis while also supporting respiratory health by easing symptoms of colds, coughs, and asthma. Additionally, it is known for its calming effect on the nervous system, providing relief from anxiety and hysteria. Traditionally, it has also been used to reduce menstrual discomfort, highlighting its diverse therapeutic benefits (Figure 2) [2,3].

The root extracts of FA, characterized by a complex blend of bioactive compounds such as ferulic acid, resin, and essential oils, have been shown to possess potent antioxidant, anti-inflammatory, and possibly antiproliferative properties [4,5]. Unlike the beneficial FA, formaldehyde is a harmful chemical used in many industries. Formaldehyde is a colorless, strong-smelling gas commonly used in industrial manufacturing and as a preservative in some products. Exposure to formaldehyde can have several toxic effects on human health, including irritation of the skin, eyes, nose, and throat and respiratory problems such as coughing, wheezing, and exacerbation of asthma. It is classified as a carcinogen linked to nasal and nasopharyngeal cancer. Additionally, formaldehyde exposure can cause dermatitis, neurological effects like headaches and dizziness, reproductive and developmental issues, sensitization leading to allergic reactions, adverse effects on the immune system, gastrointestinal irritation, and potential cardiovascular effects. It is important to minimize exposure to formaldehyde and follow safety guidelines to protect health. This makes it important to find ways to remove or reduce its presence in the environment [6,7]. The insidious nature of formaldehyde is evidenced by its documented impacts on critical organ systems, particularly the testes, kidneys, and liver, where its accumulation can lead to cellular dysfunction, tissue damage, and organ failure [6,7].

The fact that formaldehyde is harmful and FA has healing properties makes for an interesting area of study. Researchers are looking into using extracts from the roots of FA to fight the negative effects of formaldehyde. This interest is based on the ability of plant chemicals to reduce oxidative stress, which is a major problem caused by formaldehyde. These chemicals can also help control the body's defense systems at the cellular level [3,7].

This review aims to carefully examine the growing research on FA and its ability to reduce the harmful effects of formaldehyde. We focus on the testes, kidneys, and liver, which are crucial for metabolism and reproduction. Our goal is to evaluate how well FA root extracts can repair the damage caused by formaldehyde. We will look at the chemical and tissue-level evidence and discuss how FA might be used as a treatment. This review aims to present a clear and medically relevant story. By doing this, we hope to show the healing power of this old remedy and encourage more studies that could lead to new ways to protect against environmental toxins.

This article was previously posted to the Preprints.org preprint server on January 27, 2025.

## Review

FA is a veritable mosaic of bioactive compounds, each contributing to the root extract's therapeutic arsenal. At the heart of its chemical identity are sulfur-containing compounds, which not only impart the characteristic pungent odor but also play a pivotal role in its pharmacological profile. Among these, the primary active constituents include ferulic acid, umbelliprenin, asaresinotannols, and a host of essential oils, each exhibiting distinct biological activities [1,8].

Ferulic acid, a potent antioxidant, emerges as a protagonist in combating oxidative stress. Its mechanism of action is multifaceted, encompassing the scavenging of free radicals and the enhancement of the body's endogenous antioxidant defense systems. This phenolic compound has been shown to modulate several signaling pathways, including the upregulation of nuclear factor erythroid 2-related factor 2 (Nrf2), which orchestrates the expression of detoxifying enzymes [9,10].

Umbelliprenin and asaresinotannols, lesser-known but equally important constituents, have been credited with anti-inflammatory and potentially antineoplastic properties. Umbelliprenin, in particular, has been observed to inhibit lipoxygenase, thereby reducing the synthesis of pro-inflammatory mediators [2,5,11]. Meanwhile, the resin and volatile oils of FA are believed to synergize to produce its renowned antispasmodic effect, easing digestive tract spasms and mitigating gastrointestinal distress [2,5].

Historical pharmacological investigations have laid the groundwork for understanding FA's medicinal actions. Early studies highlighted its role in traditional medicine for treating respiratory ailments, an application that modern research has linked to its antiviral and expectorant activities [1,12]. Its repertoire extends to neuroprotective effects, with contemporary research exploring its potential in ameliorating symptoms and pathology associated with neurodegenerative diseases [13].

The convergence of FA's complex chemistry and its myriad pharmacological effects underscores the root extract's potential as a multifaceted therapeutic agent. It is the interplay of these compounds, both in isolation and in synergy, that underpins the root extract's capacity to confer protection against a range of pathophysiological conditions, including the insidious effects of environmental toxins such as formaldehyde [1,2,4,8].

In conclusion, the chemical composition of FA showcases nature's remarkable complexity, presenting a wealth of pharmacological potential for further investigation and therapeutic advancements. Building on the foundation of previous research, future studies hold the promise of uncovering new applications and enhancing our understanding of this ancient remedy’s significance in contemporary medicine.

Formaldehyde toxicity

Sources of Formaldehyde Exposure and Its Metabolic Pathways

Formaldehyde is a widely used volatile organic compound found in various industries, including the production of building materials and household products. It also serves as a preservative in medical laboratories and embalming fluids. Due to its widespread presence in the environment, there are growing concerns about its potential health effects. Understanding the sources of formaldehyde exposure and the biological pathways it affects is crucial. Among the common sources are building materials and home furnishings, such as carpets, upholstery, draperies, and adhesives in laminate flooring and furniture. The emission levels of formaldehyde from these sources can fluctuate based on factors like temperature, humidity, and the age of the product. Additionally, formaldehyde-releasing preservatives are used in various personal care products and medications to prevent microbial growth, including nail polish, hair gel, body washes, and certain drugs. Formaldehyde is also naturally present in some foods and can be produced in significant amounts through tobacco smoking, vaping, and the combustion of biofuels. In medical facilities and laboratories, formaldehyde is utilized for tissue preservation and as a sterilant in pathology labs, besides its use in embalming fluids [14-17].

Detailed Metabolic Pathways of Formaldehyde

Formaldehyde can directly interact with cellular components by binding to nucleic acids and proteins, creating complexes that can cause cellular dysfunction and apoptosis if not properly repaired. Additionally, formaldehyde exposure can lead to the formation of reactive oxygen species (ROS), which can cause oxidative damage to lipids, proteins, and DNA, worsening the cellular damage initiated by direct interactions with formaldehyde. Furthermore, formaldehyde can impact cellular signaling pathways, leading to abnormal cell growth and differentiation, which can contribute to the development of cancer [14,15].

In-Depth Mechanisms of Protection by FA

Compounds in FA may act as molecular chaperones, stabilizing proteins and ensuring they fold correctly [18]. This can help reduce the protein damage caused by formaldehyde. FA can also enhance the body's ability to detoxify by upregulating phase II detoxification enzymes, which help make toxins more water-soluble and easier to eliminate [14,15]. Additionally, the phytochemicals in FA can regulate the expression of genes involved in antioxidant defenses, inflammation, and apoptosis, providing a comprehensive defense against the cellular damage induced by formaldehyde [3,8,10]. By preserving mitochondrial function, FA supports adenosine triphosphate (ATP) production and cellular energy levels, essential for cell survival and repair mechanisms in response to toxic exposure [19,20]. The interaction between formaldehyde and biological systems is complex, involving direct chemical interactions with biomolecules, induction of oxidative stress, and disruption of cellular functions [14,15]. The protective effects of FA are similarly complex, addressing both the direct and indirect consequences of formaldehyde exposure [20]. This expanded understanding highlights the potential of FA not only in mitigating formaldehyde toxicity but also in offering insights into the broader application of natural compounds in combating environmental pollutants [21,22]. Further research, particularly focusing on the bioavailability, safety, and efficacy of FA extracts in humans, will be crucial in advancing our understanding and application of these protective effects.

Effects of Formaldehyde on the Testes, Kidneys, and Liver

Formaldehyde is a widely present environmental pollutant and a highly toxic substance that has been extensively researched for its harmful effects on various organ systems, including the testes, kidneys, and liver. These organs play vital roles in homeostasis, metabolism, and reproduction, making them especially susceptible to toxic exposure [23-30]. The discussion below explores the specific mechanisms through which formaldehyde impacts these organs and the resulting health consequences.

**Figure 3:**
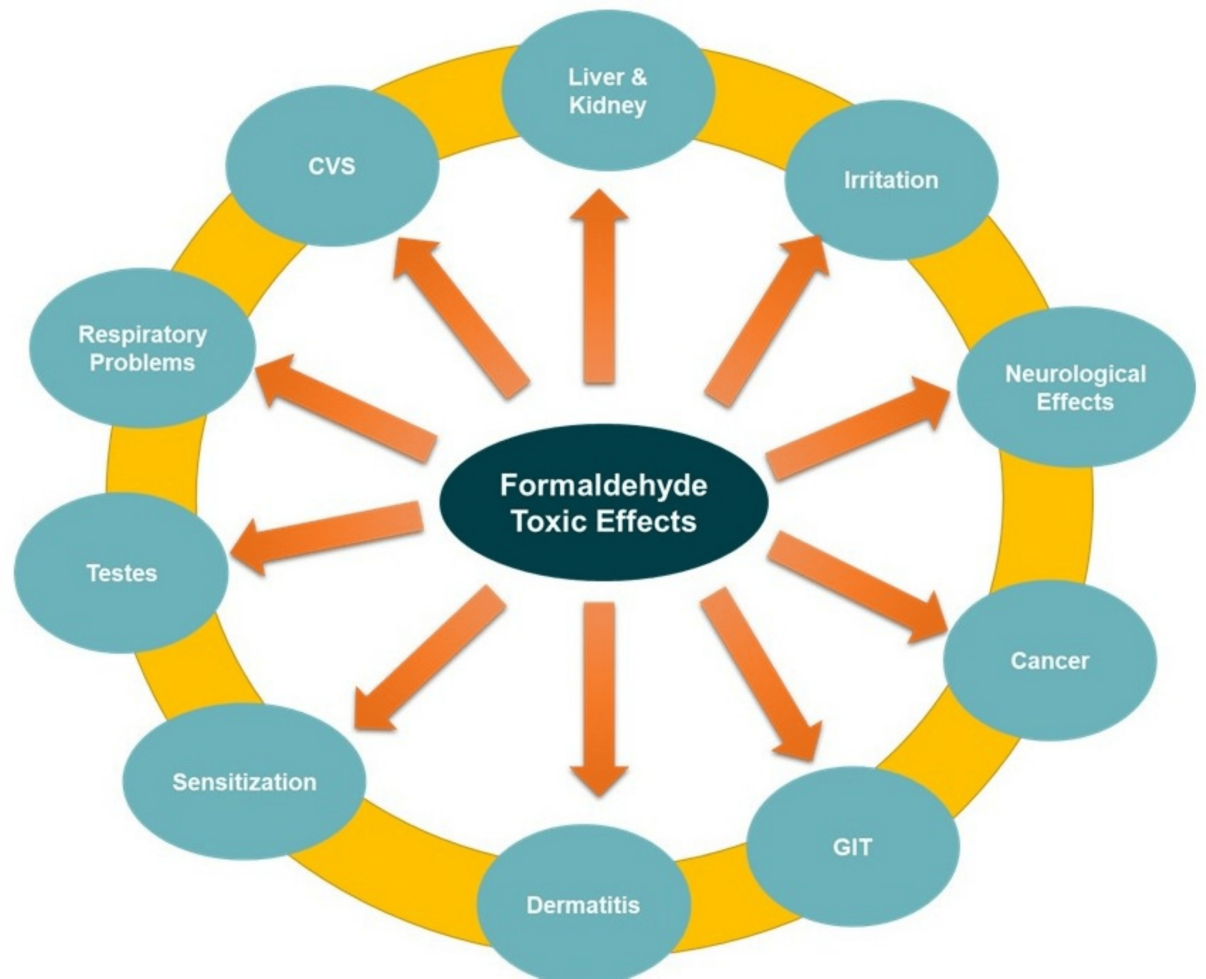
The toxic effects of formaldehyde exposure. The effects include irritation, respiratory problems, cancer, dermatitis, neurological effects, reproductive and developmental effects, sensitization, immune system effects, gastrointestinal (GIT) effects, cardiovascular (CVS) effects, and effects on the kidney and liver. The image was created by the author using PowerPoint tools (Microsoft Corporation, Redmond, WA).

Formaldehyde exposure can significantly impact male fertility and testicular function through various mechanisms. One of the primary effects is the induction of oxidative stress in the testes, where formaldehyde exposure leads to the generation of ROS that exceeds the antioxidant defense capacity of the testicular tissue. This imbalance results in oxidative damage to lipids, proteins, and DNA within sperm cells, compromising their integrity, motility, and viability. Additionally, exposure to formaldehyde can trigger apoptotic pathways in spermatogenic cells, involving the activation of caspases and the upregulation of pro-apoptotic proteins such as Bax, along with the downregulation of anti-apoptotic proteins like Bcl-2. This imbalance leads to programmed cell death, reducing sperm count and affecting fertility. Formaldehyde exposure can also disrupt hormonal balance, crucial for testicular function. It may alter the levels of gonadotropin-releasing hormone (GnRH) in the hypothalamus, luteinizing hormone (LH), and follicle-stimulating hormone (FSH) in the pituitary gland, leading to decreased testosterone production in the testes. Lower testosterone levels can affect spermatogenesis and result in reduced sexual behavior and fertility. Moreover, formaldehyde can directly affect the process of spermatogenesis, damaging spermatogonia and Sertoli cells, which are essential for the nourishment and development of sperm cells (Figure 3). This can lead to decreased sperm production and abnormal sperm morphology, further compromising male fertility [23].

The kidneys, which are responsible for filtering blood and removing waste products, are also vulnerable to the toxic effects of formaldehyde. Formaldehyde exposure can cause acute tubular necrosis, characterized by the death of cells lining the renal tubules. This damage impairs the kidney's ability to filter blood, leading to reduced renal function and potentially acute kidney injury. Additionally, formaldehyde can lead to glomerular injury, affecting the kidney's filtering units. This can result in proteinuria (excess protein in the urine), a sign of glomerular damage, and can progress to chronic kidney disease if exposure persists. Formaldehyde can also induce an inflammatory response in the kidney tissue, characterized by the infiltration of inflammatory cells and the release of pro-inflammatory cytokines. This inflammation can further exacerbate tissue damage and impair renal function. Similar to its effects on the testes, formaldehyde induces oxidative stress in the kidneys, where excessive ROS production overwhelms the antioxidant defenses, leading to lipid peroxidation, protein oxidation, and DNA damage in renal cells [23].

The liver, a central organ for metabolism and detoxification, faces significant risks from formaldehyde exposure. Formaldehyde can cause hepatocellular damage, which is evident by elevated levels of liver enzymes (alanine aminotransferase (ALT) and aspartate aminotransferase (AST)) in the bloodstream. This indicates damage to liver cells and impaired liver function. Chronic exposure to formaldehyde can lead to the accumulation of fibrous tissue in the liver (fibrosis), which can progress to cirrhosis. Cirrhosis is characterized by the replacement of healthy liver tissue with scar tissue, severely impairing liver function. As in the testes and kidneys, formaldehyde exposure results in oxidative stress in the liver. The excessive production of ROS can damage hepatocytes and disrupt liver functions, including metabolism, bile production, and detoxification processes [23]. Formaldehyde can also interfere with various metabolic functions of the liver, including glucose and lipid metabolism, leading to metabolic disturbances such as hyperglycemia and dyslipidemia. Overall, formaldehyde exposure poses significant risks to the testes, kidneys, and liver, affecting these organs' structural integrity and functional capacity [23]. The mechanisms involve oxidative stress, apoptosis, hormonal and metabolic disruptions, direct toxic effects on cellular components, and inflammation. Understanding these mechanisms is crucial for developing strategies to mitigate the adverse health effects of formaldehyde exposure [23,26].

Toxicological Mechanisms of Formaldehyde

**Figure 4:**
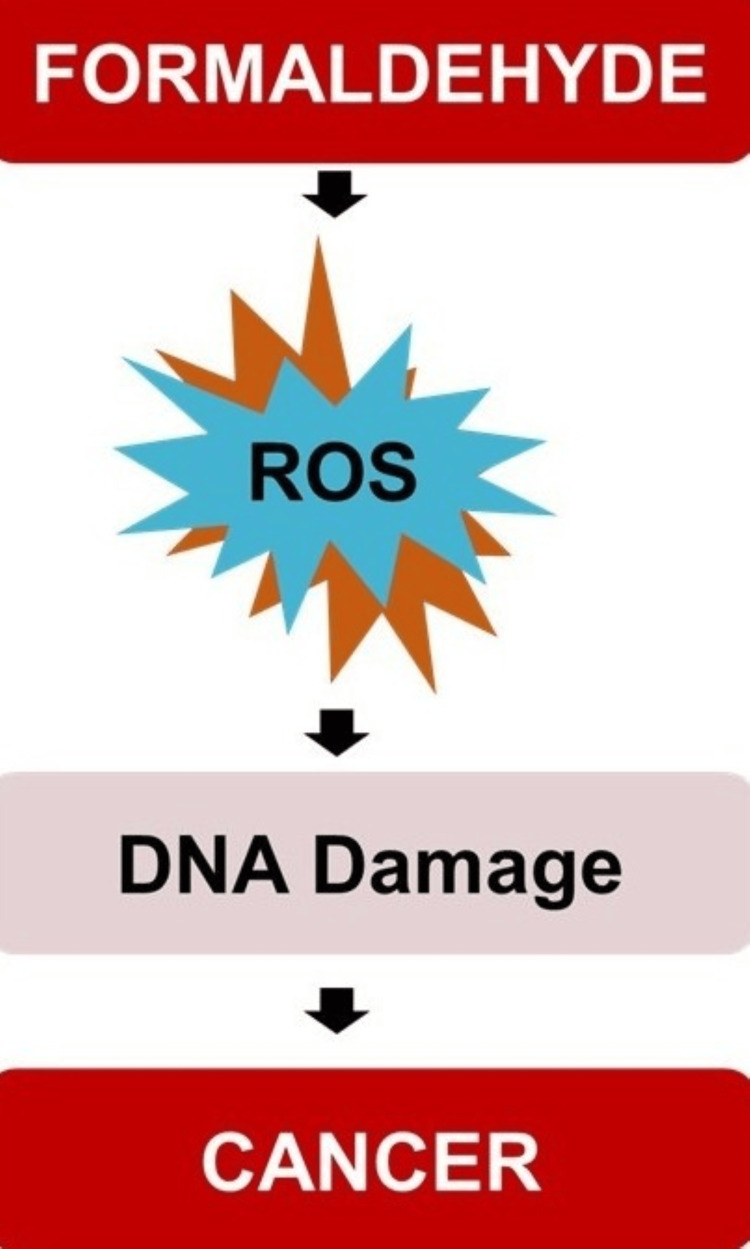
Formaldehyde exposure leads to the production of ROS, which in turn can cause DNA damage. ROS: reactive oxygen species. The image was created by the author using PowerPoint tools (Microsoft Corporation, Redmond, WA).

Recent studies have shed light on how formaldehyde affects the liver, kidney, and testes, showing complex interactions that lead to specific damage to each organ. One important study found that DNA-protein crosslinks (DPC), a key sign of damage caused by formaldehyde, were not seen in the bone marrow of rats and monkeys exposed to formaldehyde in research from the 1980s. However, earlier research from the same lab showed an increase in DPC levels in the bone marrow, liver, kidney, and testes of exposed Kunming mice. To validate these initial findings, a study subjected BALB/c mice to FA concentrations of 0, 0.5, 1.0, and 3.0 mg m^−3^ FA (eight hours per day, for seven consecutive days) via nose-only inhalation, assessing DPC accumulation in the bone marrow and additional organs. Given that oxidative stress is a plausible pathway for FA-induced harm, the authors concurrently evaluated markers of oxidative stress, including glutathione (GSH), ROS, and malondialdehyde (MDA), across various tissues such as the bone marrow, peripheral blood mononuclear cells, lung, liver, spleen, and testes. The outcomes revealed significant dose-responsive augmentations in DPC, alongside reductions in GSH, and elevations in ROS and MDA across all assessed tissues (with the exception of DPC in the lung). Notably, the bone marrow was among the tissues most significantly impacted in terms of DPC, GSH, and ROS alterations (Figure 4) [23]. This indicates systemic toxicity characterized by genotoxic effects and oxidative damage, underscoring the broad-reaching impact of formaldehyde exposure beyond the primary contact sites.

Another area of research has focused on identifying protective agents against such toxicity using fucoidan. While specific studies directly addressing formaldehyde's effects on these organs and the protective role of FA are limited, research on similar toxicological frameworks provides insights. For instance, studies on cyclophosphamide-induced organ toxicity have shown that natural compounds like fucoidan from *Laminaria japonica* can ameliorate liver and kidney injury. This is achieved by up-regulating the Nrf2/HO-1 antioxidant pathway and inhibiting the TLR4/nuclear factor-kappa B (NF-κB) inflammatory pathway, suggesting potential parallels in combating formaldehyde-induced toxicity [31].

The research highlights the protective effects of recombinant human erythropoietin (RhEPO) against oxidative stress and genotoxicity caused by the chemotherapy drugs etoposide (ETO) and methotrexate (MTX) in liver and kidney tissues. ETO and MTX are effective chemotherapeutic agents, but their use is limited due to their toxicity to healthy tissues. RhEPO, a synthetic form of the erythropoietin hormone, has been shown to protect tissues from damage. In a study with adult male Wistar rats divided into 10 groups, including control, rhEPO-only, ETO-only, MTX-only, and combinations of rhEPO with ETO or MTX, pretreatment with rhEPO was found to effectively protect liver and kidney tissues from oxidative stress caused by ETO and MTX. The administration of rhEPO led to a decrease in MDA levels, a reduction in catalase activity, and a mitigation of glutathione depletion. Additionally, rhEPO prevented DNA damage induced by the drugs, as evidenced by the comet assay. The results suggest that rhEPO, particularly at a dosage of 3000 IU/kg, has a significant protective effect against the adverse impacts of ETO and MTX on oxidative stress and genotoxicity in a biological setting [32].

The study utilized high-throughput transcriptomics in a five-day in vivo rat model to estimate benchmark dose (BMD) values for traditional toxicological endpoints in the liver, kidney, and testes. The aim was to determine if BMD values for changes in transcriptional pathways in these organs could act as proxies for BMD values associated with classical toxicological outcomes. Eighteen chemicals were selected for the investigation, including potent hepatotoxicants like DE71, perfluorooctanoic acid (PFOA), furan, and methyl eugenol, as well as others with varying levels of toxicity, such as acrylamide, α, β-thujone, ginseng, and milk thistle extract. Male Sprague Dawley rats were exposed to daily oral doses of each chemical for five consecutive days. Liver and kidney tissues were collected 24 hours after the final exposure and analyzed using high-throughput transcriptomics (HTT) on the rat S1500+ platform. Transcriptional BMD values were calculated for both liver and kidney tissues using BMDExpress 2.2, and apical BMD values for histopathological changes were computed using BMDS Wizard from existing chronic or sub-chronic toxicity studies. Interestingly, the lowest transcriptional BMDs from the five-day assays closely aligned with the lowest histopathological BMDs from conventional toxicity evaluations, typically within a factor of five. These findings suggest that HTT, when applied in a short-term in vivo model, can provide reasonable estimates of BMD values for traditional apical endpoints, potentially serving as an efficient tool to prioritize chemicals for more extensive testing. This approach highlights the value of advanced genomic techniques in understanding the dose-response relationship of toxicants like formaldehyde and assessing their risk to these critical organs [33].

In summary, the toxicological effects of formaldehyde on the liver, kidney, and testes involve mechanisms of oxidative stress, DNA-protein crosslinking, and genotoxicity. Protective strategies emerging from research, while not directly linked to FA in this context, point toward the potential efficacy of antioxidants and anti-inflammatory agents in mitigating such damage. These findings emphasize the importance of further research to validate and expand on these mechanisms and protective interventions.

Pathophysiology of organ damage induced by formaldehyde

It has been implicated in a range of deleterious health effects, particularly concerning its propensity to induce damage in critical organ systems such as the testes, kidneys, and liver. The pathophysiology of formaldehyde-induced organ damage encompasses a complex interplay of cellular and molecular mechanisms, which underpin the observed toxicological outcomes [28,29].

Testes

Emerging evidence underscores the profound effects of formaldehyde on spermatogenesis, highlighting not only oxidative stress and DNA damage but also epigenetic modifications as pivotal factors in testicular toxicity. Advanced genomic and proteomic analyses reveal that formaldehyde disrupts the expression of genes critical for sperm development and maturation, leading to aberrant spermatozoa. Additionally, recent findings suggest that formaldehyde exposure can alter histone patterns and DNA methylation status in spermatogenic cells, further compromising fertility. This disruption stems from oxidative stress, characterized by an imbalance between the production of ROS and the antioxidant defenses of the testes. Formaldehyde exposure escalates ROS production, inflicting oxidative damage on lipids, proteins, and DNA within sperm cells and Sertoli cells, the latter providing support and nutrition to developing spermatozoa.

Molecularly, formaldehyde induces DPC within testicular cells, impairing DNA replication and transcription processes vital for spermatogenesis. Furthermore, formaldehyde exposure upregulates the expression of pro-apoptotic genes and downregulates anti-apoptotic genes, triggering apoptosis in spermatogenic cells [23]. The resultant cellular loss exacerbates the decline in sperm quality and fertility potential.

Kidneys

Cutting-edge research on formaldehyde-induced nephrotoxicity has highlighted the involvement of key signaling pathways, such as the KEAP1-NRF2 pathway, in regulating the kidney's defense against oxidative stress. Transcriptomic studies have identified new biomarkers of formaldehyde exposure, offering valuable insights for early detection and potential therapeutic interventions to prevent kidney damage. Additionally, emerging research on the microbiome suggests that formaldehyde may indirectly impact renal health by influencing gut microbial composition and metabolism.

On a molecular level, formaldehyde interferes with the kidney's antioxidant response, depleting levels of key antioxidants such as GSH and reducing the activity of antioxidant enzymes like superoxide dismutase (SOD) and catalase (CAT) [23-26]. This oxidative burden is coupled with an inflammatory response, as formaldehyde exposure stimulates the renal production of pro-inflammatory cytokines, further exacerbating tissue damage.

Liver

The liver, central to metabolism and detoxification, is acutely vulnerable to formaldehyde toxicity. Formaldehyde induces hepatocellular damage, manifesting as elevated serum levels of liver enzymes, indicative of liver cell injury. The hepatotoxicity associated with formaldehyde is largely attributed to oxidative stress and inflammation. Formaldehyde exposure catalyzes the production of ROS, leading to oxidative damage to cellular lipids, proteins, and nucleic acids.

At the molecular level, formaldehyde disrupts normal hepatocellular functions by inducing alterations in gene expression related to metabolism, cell cycle regulation, and apoptosis. It impairs the liver's capacity to detoxify other substances, exacerbating its toxic effects. Additionally, formaldehyde's ability to form DPCs affects the transcriptional machinery of liver cells, further undermining hepatic function and regeneration [23-26].

In summary, the pathophysiology of formaldehyde-induced organ damage is characterized by a complex web of cellular and molecular disruptions. Oxidative stress, inflammatory responses, and direct genotoxic effects are central to the mechanisms by which formaldehyde exerts its toxicological impact on the testes, kidneys, and liver. Understanding these intricate pathways is crucial for developing therapeutic strategies to mitigate the adverse health effects associated with formaldehyde exposure.

Oxidative stress, inflammation, apoptosis, and other pathways of organ injury

The pathophysiology of organ damage induced by formaldehyde involves a complex interplay of biochemical and molecular mechanisms, where oxidative stress, inflammation, apoptosis, and other injury pathways play pivotal roles. These mechanisms, both distinct and interconnected, contribute to the toxic effects of formaldehyde on various organs, including but not limited to the liver, kidneys, and respiratory system.

Oxidative Stress

Oxidative stress is a primary mechanism of formaldehyde-induced organ damage, characterized by an imbalance between the generation of ROS and the body's ability to detoxify these reactive intermediates or repair the resulting damage. Formaldehyde exposure leads to the excessive production of ROS, such as superoxide anions, hydrogen peroxide, and hydroxyl radicals, which can attack lipids, proteins, and DNA, leading to lipid peroxidation, protein carbonylation, and nucleic acid damage. This oxidative damage compromises cellular integrity and function, contributing to the pathogenesis of organ injury [23-25]. For example, in the liver, oxidative stress can disrupt hepatocyte function and induce hepatic fibrosis, while in the kidneys, it can impair renal filtration and lead to nephropathy.

Inflammation

Formaldehyde exposure also triggers inflammatory responses, marked by the activation of various signaling pathways, including NF-κB and mitogen-activated protein kinase (MAPK) pathways, which lead to the upregulation of pro-inflammatory cytokines such as tumor necrosis factor-alpha (TNF-α), IL-1β, and IL-6. These cytokines recruit immune cells to the site of injury, exacerbating tissue damage through the release of additional ROS and proteolytic enzymes. Chronic inflammation induced by prolonged formaldehyde exposure can further drive the progression of organ damage, contributing to the development of chronic diseases such as cirrhosis in the liver or chronic kidney disease [23-25].

Apoptosis

Apoptosis, or programmed cell death, is another critical pathway through which formaldehyde induces organ damage. Formaldehyde can initiate apoptosis by damaging cellular DNA, upregulating pro-apoptotic proteins (e.g., Bax and Bad), and downregulating anti-apoptotic proteins (e.g., Bcl-2). The activation of caspases, a family of proteases essential for apoptosis, leads to the execution phase of apoptosis, characterized by cell shrinkage, chromatin condensation, and DNA fragmentation. Apoptosis plays a significant role in formaldehyde-induced toxicity, as excessive cell death can deplete cell populations in vital organs, impairing their function and regeneration capacity [23-29].

Other Pathways of Organ Injury

Beyond oxidative stress, inflammation, and apoptosis, formaldehyde exposure influences various other cellular and molecular pathways contributing to organ damage. These include the following.

Genotoxicity: Formaldehyde, a widely used industrial chemical, is recognized as a genotoxic agent with the ability to interfere with DNA integrity. It forms DNA adducts and cross-links, which are modifications where formaldehyde binds directly to DNA or links two DNA strands together. These alterations can result in mutations and chromosomal aberrations, disrupting normal cell function and potentially leading to carcinogenesis, the process by which normal cells transform into cancer cells.

The genotoxic effects of formaldehyde have been extensively studied and documented in the scientific literature. For instance, research has shown that exposure to formaldehyde can lead to an increase in DNA-protein crosslinks, a type of DNA damage associated with the formation of adducts and crosslinks. Additionally, studies have observed that formaldehyde exposure can cause chromosomal aberrations in various cell types, including human lymphocytes and mammalian cell lines [28-30].

The link between formaldehyde exposure and cancer has been a subject of significant concern. The International Agency for Research on Cancer (IARC) classifies formaldehyde as a group 1 carcinogen, indicating that there is sufficient evidence to conclude that it can cause cancer in humans. Notably, formaldehyde exposure has been associated with an increased risk of nasopharyngeal cancer, leukemia, and possibly other types of cancer.

The mechanisms underlying formaldehyde's genotoxic effects are believed to involve not only direct DNA damage but also indirect pathways such as oxidative stress and inflammation. These indirect mechanisms can further exacerbate DNA damage and contribute to the carcinogenic potential of formaldehyde [28-30].

**Figure 5:**
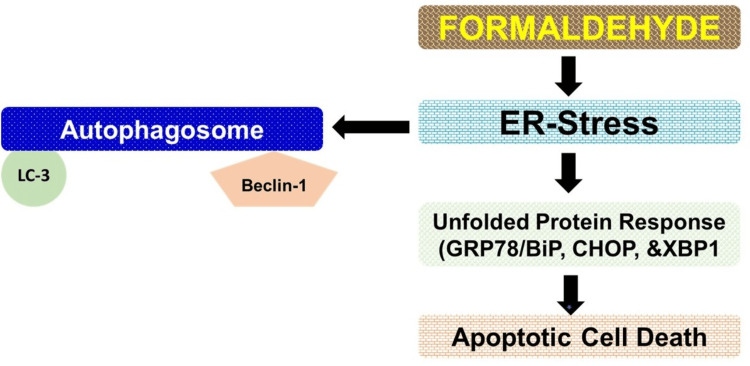
Formaldehyde exposure causes autophagy and apoptotic cell death. ER: endoplasmic reticulum; LC3: light chain 3; BAX: Bcl-2-associated X protein; BAD: Bcl-2 associated agonist of cell death; Bcl2: B-cell lymphoma 2. The image was created by the author using PowerPoint tools (Microsoft Corporation, Redmond, WA).

Formaldehyde, a ubiquitous environmental pollutant and a component of various industrial processes, has been shown to induce endoplasmic reticulum (ER) stress by disrupting protein folding within cells. This disruption leads to the activation of the unfolded protein response (UPR), a cellular mechanism aimed at restoring normal function by halting protein translation, degrading misfolded proteins, and increasing the production of molecular chaperones [34]. The UPR is a critical adaptive response that maintains ER homeostasis, but if the stress is severe or prolonged and homeostasis cannot be restored, it can trigger apoptotic cell death [35]. Formaldehyde's ability to induce ER stress and subsequently the UPR has been observed in various cellular models. For example, studies have demonstrated that formaldehyde exposure can lead to the upregulation of key UPR markers such as GRP78/BiP, CHOP, and XBP1, indicative of ER stress (Figure 5) [36-38]. The implications of formaldehyde-induced ER stress are significant, as prolonged ER stress is associated with various diseases, including neurodegenerative disorders, diabetes, and cancer [38]. Understanding the mechanisms by which formaldehyde triggers ER stress and the UPR is crucial for assessing its potential health risks and developing strategies to mitigate its adverse effects.

Autophagy is a cellular process that degrades and recycles damaged cellular components, acting as a survival mechanism under stress conditions. However, excessive or dysregulated autophagy can lead to cell death and organ injury, a phenomenon that has been observed in response to formaldehyde exposure. Formaldehyde, a common environmental pollutant and industrial chemical, has been shown to induce autophagy in various cell types. For example, studies have demonstrated that formaldehyde exposure can increase the formation of autophagosomes and the expression of autophagy-related proteins, such as LC3 and Beclin-1, in human lung epithelial cells and hepatocytes (Figure 5) [39,40]. While autophagy is initially a protective response to formaldehyde-induced stress, excessive autophagic activity can lead to autophagic cell death, also known as type II programmed cell death. This has been observed in neuronal cells exposed to formaldehyde, where prolonged autophagy contributes to neurotoxicity and cell death [39].

Moreover, dysregulated autophagy in response to formaldehyde exposure can contribute to organ injury. For instance, formaldehyde-induced autophagy has been implicated in liver injury, as evidenced by increased autophagic vacuoles and liver damage markers in animal models exposed to formaldehyde [37]. In summary, while autophagy serves as a cellular survival mechanism, excessive or dysregulated autophagy induced by formaldehyde exposure can contribute to cell death and organ injury. Understanding the balance between protective and detrimental autophagy in response to formaldehyde is crucial for assessing its health risks and developing therapeutic strategies. In summary, the pathophysiology of formaldehyde-induced organ damage is characterized by a multifaceted array of cellular and molecular mechanisms. The interplay between oxidative stress, inflammation, apoptosis, and additional pathways like genotoxicity, ER stress, and autophagy underscores the complex nature of formaldehyde toxicity. Understanding these mechanisms is crucial for developing targeted interventions to mitigate the adverse health effects associated with formaldehyde exposure.

Protective effects of FA root extracts

FA, commonly known as asafoetida, is a perennial herb widely used in traditional medicine for its vast therapeutic properties. Recent experimental studies have embarked on exploring its protective effects against the toxicity induced by formaldehyde, a known environmental and industrial pollutant. These investigations have shed light on the biochemical, molecular, and histopathological aspects of asafoetida's protective mechanisms.

Biochemical Protective Mechanisms

Several studies have demonstrated that FA root extracts possess potent antioxidant properties, which play a crucial role in mitigating oxidative stress induced by formaldehyde exposure. The root extracts are rich in compounds such as ferulic acid, umbelliprenin, and asaresinotannols, which have been shown to scavenge free radicals and enhance the activity of endogenous antioxidant enzymes such as SOD, CAT, and glutathione peroxidase (GPx). By elevating the body's antioxidant defenses, asafoetida root extracts can reduce lipid peroxidation and prevent DNA damage in organs targeted by formaldehyde toxicity, such as the liver, kidneys, and testes [1,8,41-43].

Furthermore, these extracts have been found to modulate the levels of detoxifying enzymes, contributing to the reduction of formaldehyde-induced hepatotoxicity and nephrotoxicity. By enhancing the phase II detoxification enzymes, such as glutathione S-transferase (GST), asafoetida facilitates the conjugation and subsequent elimination of toxic metabolites.

Molecular Protective Mechanisms

On a molecular level, FA has been shown to exert anti-inflammatory effects by inhibiting the NF-κB signaling pathway, which is often activated in response to formaldehyde exposure. By downregulating the expression of pro-inflammatory cytokines (TNF-α, IL-1β, IL-6) and chemokines, asafoetida root extracts can alleviate the inflammatory response and subsequent tissue damage in affected organs [42].

Moreover, experimental models have indicated that FA can modulate apoptosis pathways. The extracts have been observed to decrease the expression of pro-apoptotic proteins such as Bax and caspase-3 while upregulating anti-apoptotic proteins like Bcl-2 [43]. This modulation helps in preventing cell death and preserving the structural and functional integrity of organs exposed to formaldehyde.

Histopathological Protective Effects

Histological analyses in experimental models treated with FA following formaldehyde exposure have shown significant tissue recovery and protection. In the liver, FA treatment reduced the incidence of necrosis, fatty degeneration, and fibrosis. In the kidneys, it mitigated tubular degeneration, glomerular sclerosis, and interstitial fibrosis. Similarly, in the testes, FA preserved the architecture of seminiferous tubules and prevented germ cell loss, indicating its protective role against formaldehyde-induced testicular damage [23].

These protective effects are attributed not only to the antioxidant and anti-inflammatory properties of FA but also to its ability to enhance cellular defense mechanisms, promote detoxification, and repair damaged DNA [3]. The cumulative evidence from these experimental studies underscores the potential of FA root extracts as a natural therapeutic agent against the adverse effects of formaldehyde exposure.

The experimental evaluation of FA's protective effects against formaldehyde toxicity reveals a multifaceted mechanism of action, encompassing antioxidant, anti-inflammatory, and anti-apoptotic pathways. These findings provide a scientific basis for the traditional use of asafoetida in mitigating toxicity and suggest a promising avenue for the development of protective strategies against environmental pollutants like formaldehyde. Further research, particularly clinical trials, is necessary to validate these protective effects in humans and explore the potential of FA root extracts as a component of integrative approaches to managing chemical toxicities

Analysis of How the Root Extracts Mitigate Damage to the Testes, Kidneys, and Liver

The exploration into the protective mechanisms of FA root extracts against formaldehyde-induced toxicity reveals a fascinating interplay of biochemical and cellular defenses that safeguard the testes, kidneys, and liver from damage. These root extracts, esteemed in traditional medicine for their therapeutic virtues, have been scientifically scrutinized to unveil their role in countering the deleterious effects of environmental pollutants like formaldehyde. The elucidation of these protective mechanisms underscores a sophisticated synergy of antioxidant, anti-inflammatory, and anti-apoptotic actions facilitated by the phytochemical constituents of FA.

The strong antioxidant properties of FA root extracts play a key role in their protective effects. These extracts contain phytochemicals like ferulic acid, umbelliprenin, and asaresinotannols, which help defend against oxidative stress, a major cause of organ damage from formaldehyde exposure. These compounds scavenge ROS, thereby mitigating lipid peroxidation and preserving the integrity of cellular membranes in the testes, kidneys, and liver. Moreover, they bolster the endogenous antioxidant defense system, enhancing the activities of SOD, CAT, and GPx [44]. This concerted antioxidant action prevents the oxidative deterioration of cellular components, ensuring the maintenance of organ function and vitality.

Inflammation plays a pivotal role in the pathophysiology of formaldehyde-induced organ damage, with pro-inflammatory cytokines exacerbating tissue injury. FA root extracts exhibit a remarkable capacity to modulate the inflammatory cascade, primarily by inhibiting the NF-κB signaling pathway. This modulation results in the downregulation of pro-inflammatory mediators such as TNF-α, IL-1β, and IL-6, curtailing the inflammatory insult to the testes, kidneys, and liver [43-45]. By dampening the inflammatory response, these extracts alleviate the histopathological changes associated with formaldehyde exposure, preserving organ architecture and function.

Apoptosis, or programmed cell death, is a critical mechanism through which formaldehyde induces organ toxicity. The root extracts of FA exert a protective influence by modulating the apoptotic pathways. They enhance the expression of anti-apoptotic proteins such as Bcl-2 while suppressing the expression of pro-apoptotic proteins, including Bcl-2-associated X protein (Bax) and caspase-3 [45]. This regulation of apoptotic mediators prevents the excessive loss of parenchymal cells in the testes, kidneys, and liver, thwarting the decline in organ functionality attributable to cell death.

FA root extracts contribute to the detoxification of formaldehyde and its metabolites by upregulating phase II detoxification enzymes, such as GST. This enzymatic upregulation facilitates the conjugation and subsequent excretion of toxic substances, reducing their bioaccumulation and mitigating their toxic impact on organ tissues.

The protective effects of FA root extracts against formaldehyde-induced damage in the testes, kidneys, and liver are emblematic of their multifaceted pharmacological actions. Through the augmentation of antioxidant defenses, modulation of inflammatory responses, regulation of apoptotic pathways, and enhancement of detoxification processes, these extracts offer a promising natural therapeutic strategy for mitigating the adverse health effects of formaldehyde exposure. The elucidation of these mechanisms not only validates the traditional use of FA in herbal medicine but also opens new avenues for the development of integrative approaches to combat environmental toxicities.

The extracts of FA, a plant revered in traditional medicine, possess a fascinating array of pharmacological properties, including antioxidant, anti-inflammatory, and anti-apoptotic effects [44]. These properties confer a protective advantage against various forms of cellular and organ damage, particularly those induced by environmental toxins like formaldehyde. A deeper dive into each of these properties reveals the intricate mechanisms by which FA exerts its therapeutic effects.

The antioxidant capacity of FA extracts is central to their protective role against oxidative stress, a condition characterized by an imbalance between the production of ROS and the body's ability to neutralize these harmful compounds. The extracts are rich in potent phytochemicals such as ferulic acid, umbelliprenin, and asaresinotannols, which directly scavenge free radicals, thereby reducing the burden of ROS. Additionally, these compounds can chelate metal ions, further diminishing the catalytic decomposition of hydrogen peroxide into more reactive radicals [42-44].

On a cellular level, FA extracts upregulate the expression and activity of endogenous antioxidant enzymes, including SOD, CAT, and GPx. SOD converts superoxide radicals into hydrogen peroxide, CAT then decomposes hydrogen peroxide into water and oxygen, and GPx reduces hydrogen peroxide by converting reduced glutathione into its oxidized form. This enzymatic cascade fortifies the cell's defense against oxidative damage, safeguarding DNA, proteins, and lipids from oxidative modifications that can lead to cell dysfunction and death.

Inflammation, a biological response to cellular damage or pathogens, can become deleterious when chronic or excessive. FA extracts exhibit significant anti-inflammatory effects by modulating various components of the inflammatory pathway. These extracts inhibit the activation of NF-κB, a transcription factor that plays a pivotal role in the expression of pro-inflammatory cytokines such as TNF-α, IL-1β, and IL-6. By suppressing NF-κB translocation to the nucleus, FA extracts prevent the transcription of genes responsible for the inflammatory response, thereby reducing the production of inflammatory mediators and attenuating the inflammatory process.

Moreover, these extracts can interfere with other inflammatory signaling pathways, such as the MAPK pathway, further contributing to their anti-inflammatory efficacy. By dampening the inflammatory cascade, FA extracts mitigate the tissue damage and pathological alterations associated with chronic inflammation.

Apoptosis, a form of programmed cell death, is essential for maintaining cellular homeostasis but can lead to tissue damage if dysregulated. FA extracts possess anti-apoptotic properties that help maintain cell viability under stress conditions. These extracts modulate the expression of key proteins involved in the apoptotic pathway, including upregulation of anti-apoptotic proteins like Bcl-2 and downregulation of pro-apoptotic proteins such as Bax and caspase-3.

By altering the balance of pro- and anti-apoptotic proteins, FA extracts prevent the activation of caspases, the executioners of apoptosis, thereby inhibiting cell death. This protective mechanism is particularly beneficial in preventing the loss of critical cells in response to toxic insults, such as those induced by formaldehyde exposure, ensuring the preservation of organ function and structure.

The antioxidant, anti-inflammatory, and anti-apoptotic properties of FA extracts underscore their potential as therapeutic agents against a spectrum of pathological conditions driven by oxidative stress, inflammation, and apoptosis. Through a multifaceted approach that combines the scavenging of free radicals, modulation of inflammatory and apoptotic pathways, and enhancement of the body's natural defense mechanisms, these extracts offer a promising avenue for the development of novel treatments aimed at mitigating cellular and organ damage.

Methodological approaches

Experimental Designs Used in the Studies

The exploration of FA root extracts' protective properties against formaldehyde-induced toxicity encompasses a diverse array of experimental designs. Predominantly, rodent models, including rats and mice, serve as the primary subjects due to their physiological resemblance to humans and well-documented responses to toxicants like formaldehyde [43]. These studies typically involve exposing animals to formaldehyde via inhalation or injection, simulating real-world environmental and occupational exposure scenarios. The concentration and duration of formaldehyde exposure are meticulously calibrated to assess both acute and chronic effects, providing insights into the short-term and long-term impacts of formaldehyde toxicity.

In parallel, FA root extracts are administered to the animals in various ways, tailored to the study's objectives. The extracts may be given before, during, or after formaldehyde exposure, with the administration route (oral, intraperitoneal, or subcutaneous) and timing chosen based on the desired therapeutic outcome and the pharmacokinetic characteristics of the extracts. Control groups are included in these studies to differentiate the protective effects of the extracts from the toxic effects of formaldehyde. Furthermore, dose-response analyses are often conducted to identify the optimal protective dose of FA extracts, providing valuable data for potential therapeutic applications [46-48].

Methods of extract administration

Oral Administration

This is the most common method used in animal studies, where the extracts are given orally, either in solution form or mixed with food, to simulate dietary supplementation. This route is preferred for its ease of administration and relevance to potential human consumption [49].

Intraperitoneal Injection

In some cases, the extracts are administered through intraperitoneal injection to ensure rapid absorption and distribution of the active compounds. This route is particularly useful for studying the acute effects of the extracts and their pharmacokinetic properties [47].

Subcutaneous Injection

Although less common, subcutaneous injection is used in certain studies to assess the local effects of the extracts on specific organs or tissues. This route can provide valuable insights into the extracts' mechanism of action and potential therapeutic applications [50].

The choice of administration route is influenced by various factors, including the bioavailability of the active compounds in the extracts, the desired onset speed of the protective effects, and the feasibility of administering repeated doses in studies involving chronic exposure. Through these methodological approaches, researchers aim to elucidate the protective mechanisms of FA root extracts and their potential as natural therapeutic agents against formaldehyde-induced toxicity.

Doses of FA Extracts

The determination of the optimal dose of FA extracts is crucial for maximizing their protective effects while minimizing potential side effects. Doses vary significantly across studies, depending on the specific extract used, the route of administration, and the animal model. Commonly, doses range from low (e.g., 50-100 mg/kg body weight) to high (e.g., 300-500 mg/kg body weight) concentrations. The choice of dose is often guided by preliminary dose-response studies, which help identify the most effective concentration for mitigating formaldehyde-induced toxicity. It is important to note that the active constituents of FA, such as ferulic acid and umbelliprenin, have different potencies, which can influence the optimal dose for each extract [51,52].

Duration of Treatment

The duration of treatment with FA extracts is another critical factor that can affect the protective outcomes. Treatment duration can range from acute (e.g., a single dose or a few days) to chronic (e.g., several weeks to months) administration. Acute treatments are typically used to assess the immediate protective effects of the extracts against formaldehyde-induced damage, while chronic treatments are employed to evaluate the long-term benefits and potential side effects of sustained extract consumption. The choice of treatment duration is often based on the study's objectives and the nature of formaldehyde exposure (acute vs. chronic) [21,22,42].

Methods for evaluating organ function and pathology

Biochemical Assays

These assays measure specific biomarkers in blood, urine, or tissue samples to evaluate organ function. For example, liver function is assessed by measuring serum levels of ALT and AST, while kidney function is evaluated by measuring blood urea nitrogen (BUN) and creatinine levels. Antioxidant enzyme activities (e.g., SOD, CAT, and GPx) and markers of oxidative stress (e.g., malondialdehyde (MDA)) are also quantified to assess the antioxidant effects of the extracts [21,22,42].

Histological Examination

Histological analysis of organ tissues provides insights into the structural changes induced by formaldehyde exposure and the protective effects of FA extracts. Tissue sections are stained with hematoxylin and eosin (H&E) or other specific stains and examined under a microscope for signs of inflammation, necrosis, fibrosis, and other pathological alterations [20-24,42].

Molecular Techniques

Techniques such as real-time polymerase chain reaction (RT-PCR), Western blotting, and immunohistochemistry are used to assess the expression levels of genes and proteins involved in oxidative stress, inflammation, apoptosis, and other relevant pathways. These molecular analyses provide mechanistic insights into how FA extracts exert their protective effects [20-25,42].

In conclusion, the determination of optimal doses, appropriate treatment durations, and the use of comprehensive methods for evaluating organ function and pathology are essential components of studies investigating the protective effects of FA extracts against formaldehyde-induced toxicity. These factors collectively contribute to a better understanding of the therapeutic potential of FA and its application in mitigating the adverse effects of environmental pollutants.

Discussion

FA, a traditional medicinal herb, has garnered attention for its potential to mitigate the adverse effects of formaldehyde, a pervasive environmental toxin. This review delves into the protective properties of FA root extracts against formaldehyde-induced toxicity in vital organs such as the liver, kidneys, and testes. The focus is on understanding the underlying mechanisms of protection, including antioxidant and anti-inflammatory activities, and evaluating the efficacy of different extract preparations, dosages, and treatment durations.

The therapeutic potential of FA is attributed to its rich array of bioactive compounds, including ferulic acid, umbelliprenin, and asaresinotannols. These compounds are known for their antioxidant properties, which play a crucial role in combating oxidative stress, a key factor in formaldehyde-induced organ damage. Additionally, the anti-inflammatory effects of these phytochemicals contribute to the protective action of FA extracts.

Formaldehyde exposure is associated with various health risks, including damage to critical organ systems. The liver, kidneys, and testes are particularly vulnerable to formaldehyde-induced toxicity due to their roles in metabolism, detoxification, and reproduction. The toxicological mechanisms of formaldehyde involve direct interactions with cellular components, induction of oxidative stress, and disruption of cellular signaling pathways.

The protective effects of FA root extracts against formaldehyde toxicity are multifaceted. The extracts exhibit antioxidant properties, enhancing the body's defense against oxidative stress by scavenging free radicals and boosting the activity of endogenous antioxidant enzymes. Anti-inflammatory actions are mediated through the inhibition of pro-inflammatory cytokine production and modulation of signaling pathways such as NF-κB. Additionally, the extracts exert anti-apoptotic effects, regulating the balance between pro- and anti-apoptotic proteins to prevent cell death.

Various experimental studies have demonstrated the protective effects of FA root extracts against formaldehyde-induced organ damage. Biomarker assays measure liver (ALT, AST) and kidney (BUN, creatinine) function, along with antioxidant enzyme activities (SOD, CAT, GPx) and oxidative stress markers (MDA). Histological analysis examines tissue changes caused by formaldehyde exposure using staining techniques to identify inflammation, necrosis, and fibrosis. Molecular methods, including RT-PCR, Western blotting, and immunohistochemistry, analyze gene and protein expression related to oxidative stress, inflammation, and apoptosis, providing insights into the protective mechanisms of FA extracts [42-44].

The optimal dose and duration of treatment with FA extracts are crucial factors that influence their protective efficacy. Experimental studies have employed a range of doses and treatment durations to identify the most effective regimens. The evaluation of organ function and pathology involves a combination of biochemical assays, histological examination, and molecular techniques, providing a comprehensive assessment of the protective effects of the extracts.

The synthesis of findings from various studies on the protective effects of FA root extracts against formaldehyde-induced toxicity reveals a consistent pattern of antioxidant, anti-inflammatory, and anti-apoptotic activities across different organ systems, particularly the testes, kidneys, and liver [22-28,42-44]. These studies consistently demonstrate the ability of FA extracts to mitigate oxidative stress, reduce inflammation, and prevent cell death, leading to the preservation of organ structure and function [22-28,42-44]. However, discrepancies in the optimal doses and treatment durations highlight the need for further research to standardize these parameters for maximum therapeutic efficacy.

A comparative analysis of the effectiveness of FA root extracts on the testes, kidneys, and liver indicates that while the extracts exhibit protective effects across all three organs, the magnitude and specific mechanisms of protection may vary. For instance, the extracts show pronounced anti-inflammatory effects in the liver by modulating the NF-κB pathway, while in the testes, the antioxidant properties play a more significant role in preserving spermatogenesis [22-28,42-44]. This variability underscores the complexity of the extracts' pharmacological actions and the organ-specific nature of formaldehyde-induced toxicity.

The potential for translation from animal models to human scenarios is promising, given the consistent protective effects observed in rodent studies. However, the challenge lies in extrapolating these findings to human contexts, considering the differences in physiology, metabolism, and exposure levels between humans and animal models [19-22]. Rigorous, controlled clinical trials are essential to validate the therapeutic viability of FA in humans and to determine the appropriate dosages and treatment regimens for effective protection against formaldehyde toxicity [51-53].

Challenges and limitations

The exploration of FA’s protective effects against formaldehyde-induced organ damage presents several challenges and limitations that need to be addressed to fully understand its therapeutic potential. One of the primary challenges is the extrapolation of findings from animal models to human scenarios. While studies conducted on rodents provide valuable insights, there are physiological and metabolic differences between species that can affect the applicability of these results to humans. Additionally, the exposure levels and routes of administration used in animal studies may not accurately reflect human exposure to formaldehyde, making it difficult to translate these findings into practical human applications.

Another significant limitation is the variability in the optimal doses and treatment durations of FA extracts across different studies. This inconsistency poses a challenge in determining standardized therapeutic regimens for maximum efficacy. The complexity of the bioactive compounds present in FA further complicates this issue, as isolating and characterizing the specific compounds responsible for the protective effects is a daunting task. Ensuring the consistency and reproducibility of extract preparations used in various studies is also a concern.

The absence of rigorous, controlled clinical trials is a major limitation highlighted in the paper. Without such trials, it is difficult to ascertain the safety, efficacy, and optimal administration protocols of FA extracts for protecting against formaldehyde-induced organ damage in humans. Moreover, the potential side effects or adverse reactions associated with the use of FA extracts need to be thoroughly evaluated, especially in the context of chronic exposure to formaldehyde.

Furthermore, the potential interactions between FA extracts and other medications or supplements have not been extensively studied. Understanding these interactions is crucial for the safe and effective use of FA as a protective agent against formaldehyde toxicity. In conclusion, while the protective effects of FA against formaldehyde-induced organ damage are promising, further research, including clinical trials, is needed to address these challenges and limitations and establish its therapeutic potential in human scenarios.

## Conclusions

The exploration of FA's protective effects against formaldehyde-induced organ damage, particularly in the liver, kidneys, and testes, presents a promising avenue for developing natural therapeutic interventions against environmental toxins. The comprehensive analysis of existing literature highlights the potent antioxidant, anti-inflammatory, and anti-apoptotic properties of FA root extracts, which collectively contribute to mitigating the deleterious effects of formaldehyde exposure. The intricate pathophysiological mechanisms underlying formaldehyde toxicity have been elucidated, emphasizing the role of oxidative stress, inflammation, and apoptosis in organ damage.

Despite the promising findings, several challenges and limitations, such as dose optimization, long-term effects, and translation to human scenarios, need to be addressed in future research. Rigorous clinical trials are essential to validate the efficacy and safety of FA root extracts in humans and to establish standardized therapeutic regimens. Additionally, a deeper understanding of the molecular targets and signaling pathways involved in the protective effects of the extracts is crucial for their therapeutic application. In conclusion, FA root extracts offer a beacon of hope for individuals afflicted by formaldehyde toxicity. The potential of this ancient remedy in modern medicine is vast, and further research is warranted to unlock its full therapeutic potential and pave the way for novel protective agents against environmental toxins.
